# Variations in the immune and metabolic response of proactive and reactive *Sparus aurata* under stimulation with *Vibrio anguillarum* vaccine

**DOI:** 10.1038/s41598-018-35863-w

**Published:** 2018-11-26

**Authors:** R. Vargas, J. C. Balasch, I. Brandts, F. Reyes-López, L. Tort, M. Teles

**Affiliations:** 1 Programa Inserción SENACYT-Universidad de Panamá, Extensión Universitaria de Aguadulce, Aguadulce, Panama; 2 Department of Cell Biology, Physiology and Immunology, Universitat Autònoma de Barcelona, 08193 Barcelona, Spain; 3 CIIMAR-Interdisciplinary Centre of Marine and Environmental Research, Terminal de Cruzeiros do Porto de Leixões, Avenida General Norton de Matos, S/N, 4450-208 Matosinhos, Portugal

## Abstract

Environmental insults, such as exposure to pathogens, modulate the behavioural coping style of animals to stressors, and repeated exposure to stressful environments may lead to species-specific infection phenotypes. To analyse the influence of stress behavioural phenotypes on immune and metabolic performance, gilthead sea bream (*Sparus aurata* L.) were first screened for proactive and reactive coping styles. Once characterized, both behavioural phenotypes fish groups were bath vaccinated with bacterin from *Vibrio anguillarum*, an opportunistic widespread pathogen of fish. Gills and liver were sampled at 0 (control group), 1, 3 and 7 days post-vaccination. Immune-, oxidative stress- and metabolic-related transcripts (*il1β*, *tnfα*, *igm*, *gpx1*, *sod*, *cat*, *lpl*, *ghr1* and *ghr2*), metabolic endpoints (glucose, cholesterol and triglycerides), hepatic health indicators (aspartate aminotransferase, alanine transaminase and alkaline phosphatase), oxidative stress status (esterase activity, total antioxidant capacity and total oxidative status) and stress biomarkers (cortisol) were determined. Present results indicate that screening for coping styles in the gilthead sea bream segregated the two distinct phenotypes as expected: proactive and reactive. Results also indicate that under bath vaccination proactive fish show high immune response and lower metabolism, whereas reactive fish show low immune and higher metabolic responses.

## Introduction

Individual variations in behavioural responses of animals exposed to environmental challenges have been studied in vertebrates since the late 1980’s^1^. These distinct behavioural strategies among individuals from the same population have been described in literature using distinct terms, such as individuality, animal personality^2^, behavioural syndromes^3^, temperaments^4^, or coping styles^5^. All of these terms ultimately seek to describe consistent links between traits (behavioural, physiological, or both) performance and fitness, and have also been observed in fish^6^. One of the most widely accepted terms to describe these differences in behaviour is “coping styles’, defined by Koolhaas *et al*. (1999) as ‘a coherent set of behavioural and physiological stress responses, consistent over time and characteristic to a certain group of individuals’. In fish, studies regarding coping strategies have been conducted in several farmed species including *Salmo salar*^7^, *Oncorhynchus mykiss*^8^, *Cyprinus carpio*^9^, *Sparus aurata*^10^ and *Dicentrarchus labrax*^11^, all of these species of high economic value. A useful operative classification separates fish behaviour in two opposing stress-coping styles: proactive (adrenaline based, active coping or ‘fight-flight’) and reactive (cortisol based, passive coping or’conservation-withdrawal’). True proactive individuals display behavioural phenotypes based on active scape from stressors, high feed efficiency and motivation after an environmental change, dominance in aggressive encounters and low sensitivity to environmental stressors^12–15^. Physiologically, proactive fish exhibit traits such as both low basal cortisol levels and low cortisol reactivity in stressful situations, low hormonal modulation, high oxygen consumption during stress and intense immune responses^12,15–18^. These traits may anticipate, as some authors suggest, that proactive fish will fare better in stable, plentiful, high-density environments, while reactive individuals will thrive best in environments with sparse, unpredictable resources, and low animal densities^3^. Differences in coping styles may also influence disease resistance, growth performance, metabolic adjustments and, ultimately, fish welfare under intensive aquaculture practice^1^, where high stock densities facilitate the spreading of bacterial and viral pathogens.

In this study, we aimed to evaluate how fish with different coping styles respond to the stress of vaccination over time. For that purpose, gilthead sea bream (*S. aurata*) were firstly screened for coping styles and posteriorly subjected to a stressor that may occur under aquaculture conditions, i.e., bath vaccination against *Vibrio anguillarum*, an opportunistic Gram-negative bacterium that leads to epidemic vibriosis in more than 90 aquatic species worldwide^19^. The levels of gene transcripts associated with immune response (interleukin 1β – *il1β*, tumor necrosis factor-α – *tnfα*, immunoglobulin M - *igm*) and oxidative status (glutathione peroxidase 1 – *gpx1*, superoxide dismutase –*sod*, catalase –*cat*) were determined in gills. Biochemical indicators of oxidative status (esterase activity –EA, total antioxidant status –TAC, total oxidant status –TOS) were also assessed in gills. In the liver, changes in transcript abundance of *il1β*, *tnfα*, *igm*, *gpx1*, *sod*, *cat*, and mRNAs related with metabolism (lipoprotein – *lpl*, growth hormone receptors type I and II – *ghr1* and *ghr2*) were determined. Biochemical indicators of lipid metabolism (cholesterol, triglycerides), carbohydrate metabolism (glucose), hepatic health indicators (aspartate aminotransferase – AST, alanine transaminase – ALT, alkaline phosphatase – ALP) and oxidative status (EA, TAC and TOS) were assessed in liver. Cortisol and glucose were measured in plasma as indicators of the stress response.

## Results

### Behavioural screening

**Figure 1:**
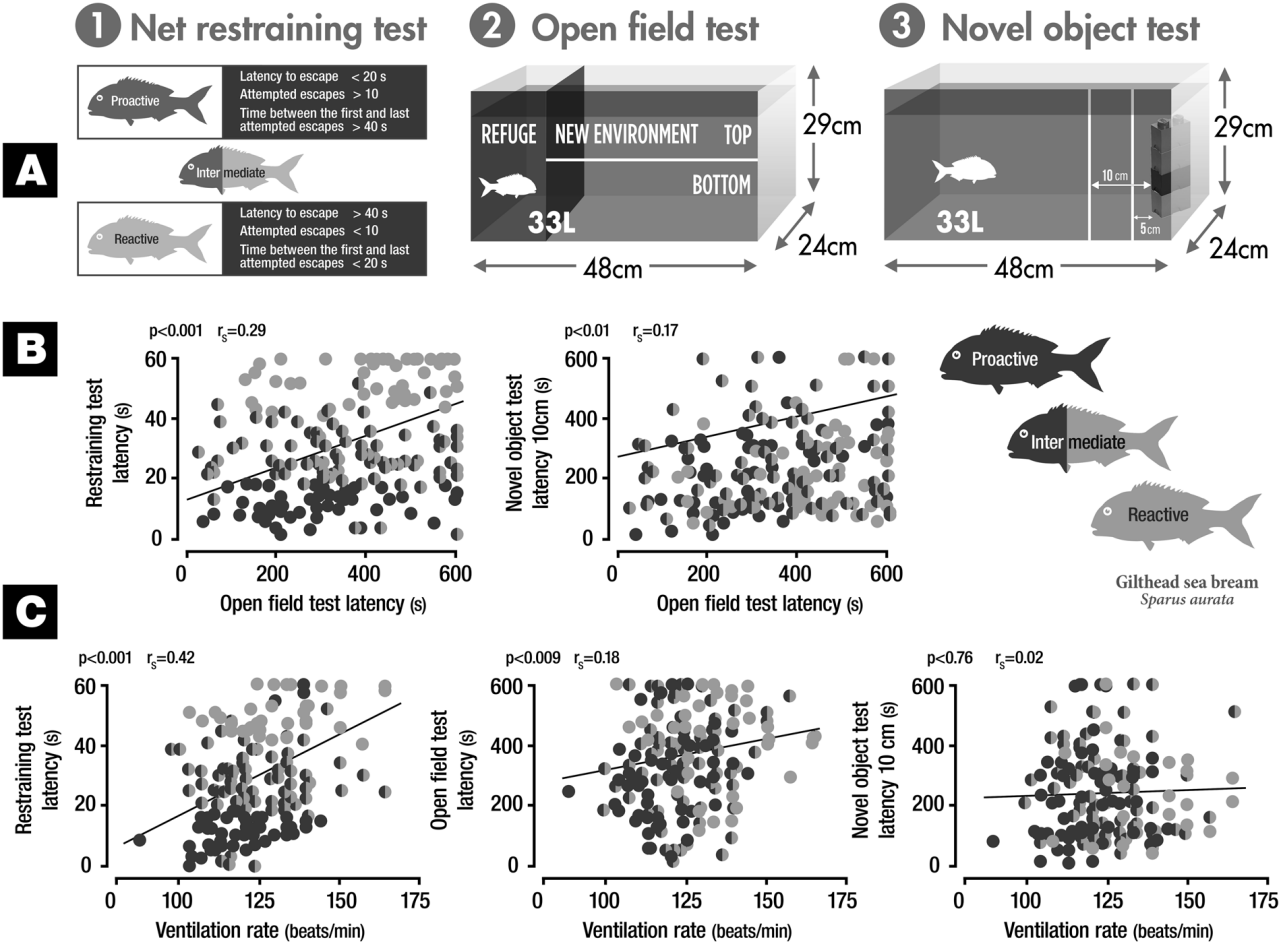
Outline of behavioural tests and behavioural screening for proactive and reactive phenotypes in *S. aurata*.

Restraining, open field and new object tests (Fig. 1A) were suitable to separate *S. aurata* according to proactive, intermediate and reactive behavioural phenotypes. A good correlation was found between assessed behavioural tests. Fish who presented shorter latency to reach the tank’s upper half in the open field test also showed lower latency to attempt the first escape in the net restriction test (r_s_ = 0.29, p < 0.00, Fig. 1B), as well as smaller/shorter latency to enter the 10 cm area in the new object test (r_s_ = 0.17, p = 0.01, Fig. 1B). Ventilation rate and latency were correlated to first attempted escape in net restriction test (r_s_ = 0.42, p < 0.001, Fig. 1C). Individuals that showed lower ventilation rate also presented lower latency to reach the tank’s upper half in the open field test (r_s_ = 0.18, p = 0.009, Fig. 1C). No correlation was found between ventilation rate and latency to enter the 10 cm area in the new object test (r_s_ = 0.02, p = 0.76, Fig. 1C).

### Gene expression and biochemical endpoints in gills

**Figure 2:**
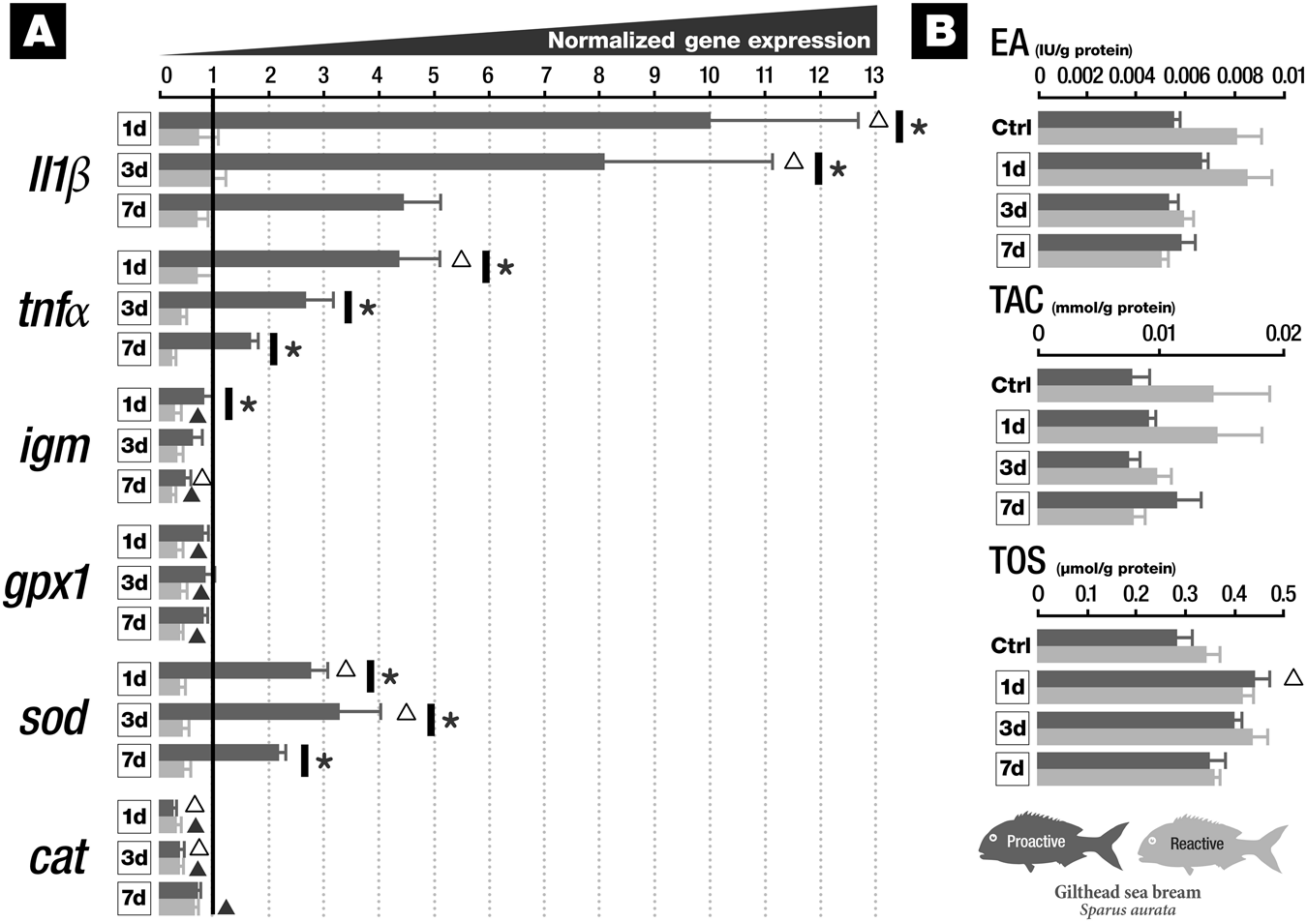
Gill responses of proactive and reactive fish exposed to *Vibrio anguillarum* bacterin at 1, 3 and 7 days post-vaccination (**A**) Expression profiles of immune- (*il1β*, *tnfα* and *igm*) and oxidative stress-related (*gpx1*, *sod* and *cat*) gene transcripts. (**B**) Biochemical endpoints (EA, TAC and TOS) (see the text for abbreviations). *indicates significant differences between proactive and reactive fish (p < 0.05). ∆ indicates significant differences between proactive and control fish (p < 0.05). ▴ indicates significant differences between reactive and control fish (p < 0.05). Data are presented as mean ± SD.

With respect to genes belonging to the immune system (Fig. 2A), it was observed that *il1β* presented increased mRNA abundance in proactive groups 1 and 3 days after vaccination when compared with both control and reactive groups in gills. The mRNA abundance of *tnfα* increased in the proactive group 1 day after vaccination when compared to the control group. Furthermore, proactive fish presented increased mRNA levels of *tnfα* for all sampling periods when compared to the reactive groups. The mRNA levels of *igm* significantly decreased in proactive groups compared to the control group, and were decreased in the reactive group 1, 3 and 7 days post-vaccination (dpv) compared to control as well. Moreover, at day 1 after vaccination *igm* mRNA levels were also significantly lower in reactive fish compared to proactive fish. Concerning oxidative status mRNAs, we observed a significant decrease in *gpx1* in reactive fish at 1, 3 and 7 dpv when compared to control; *sod* mRNA levels were significantly increased in proactive fish 1 and 3 dpv when compared to the control group. Moreover, *sod* mRNA levels were significantly decreased in reactive fish after 1, 3 and 7 dpv when compared to proactive fish. The transcript *cat*, presented decreased mRNA levels in proactive fish at day 1 and 3 post vaccination compared to the control group as well as in reactive fish have after all exposure sampling periods when compared to the control group. For all the other tested conditions, no changes were observed. Concerning biochemical endpoints (Fig. 2B), TOS presented an increased activity for proactive group on day 1 after vaccination. TOS activity was at control level for all the other conditions. EA and TAC were unchanged in gills.

### Gene expression and biochemical endpoints in liver

**Figure 3:**
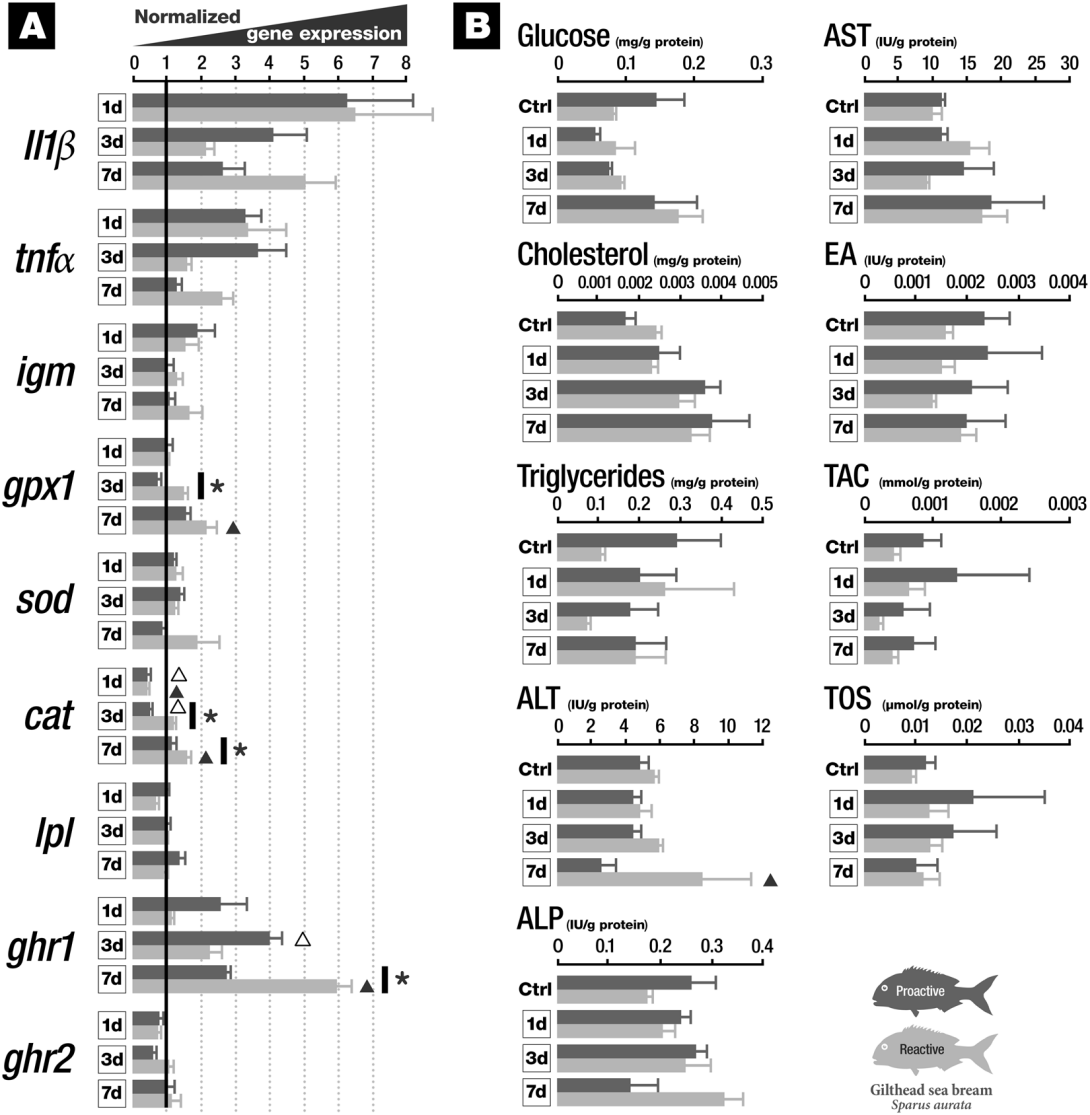
Liver responses of proactive and reactive fish exposed to *Vibrio anguillarum* bacterin at 1, 3 and 7 days post-vaccination (**A**) Expression profiles of immune- (*il1β*, *tnfα* and *igm*), oxidative stress- (*gpx1*, *sod* and *cat*) and metabolic-related (*lpl*, *ghr1* and *ghr2*) transcripts. (**B**) Biochemical endpoints (glucose, cholesterol, triglycerides, ALT, ALP, AST, EA, TAC and TOS) (see the text for abbreviations). *indicates significant differences between proactive and reactive fish (p < 0.05). ∆ indicates significant differences between proactive and control fish (p < 0.05). ▴ indicates significant differences between reactive and control fish (p < 0.05). Data are presented as mean ± SD.

Immune-related transcripts, namely *il1β*, *tnfα* and *igm* presented mRNA abundance similar to that of the control group in the liver (Fig. 3A). No changes were found when comparing proactive and reactive groups of fish. With respect to mRNAs associated with antioxidant function, an increase in mRNA levels of *gpx1* in reactive fish 7 days after vaccination was observed when compared to the control group. Transcript levels of *gpx1* exhibit an increase in reactive fish in comparison to proactive fish, 3 days post vaccination. Regarding transcripts related to metabolism, an increase in mRNA levels of *ghr1* in proactive group 3 days after vaccination compared to the control group was observed, as well as in reactive group at 7 dpv compared to the control group. Furthermore, mRNA levels of *ghr1* were significantly increased in reactive fish 7 days after vaccination when compared to proactive fish. Levels of *lpl* and *ghr2* mRNAs were unaltered. For all the other conditions, the studied mRNAs presented similar levels to the control group. With regard to the biochemical endpoints (Fig. 3B), an increase in ALT levels in liver was found. All other studied endpoints were unaltered in liver of *S. aurata*.

### Plasma cortisol and glucose

**Figure 4:**
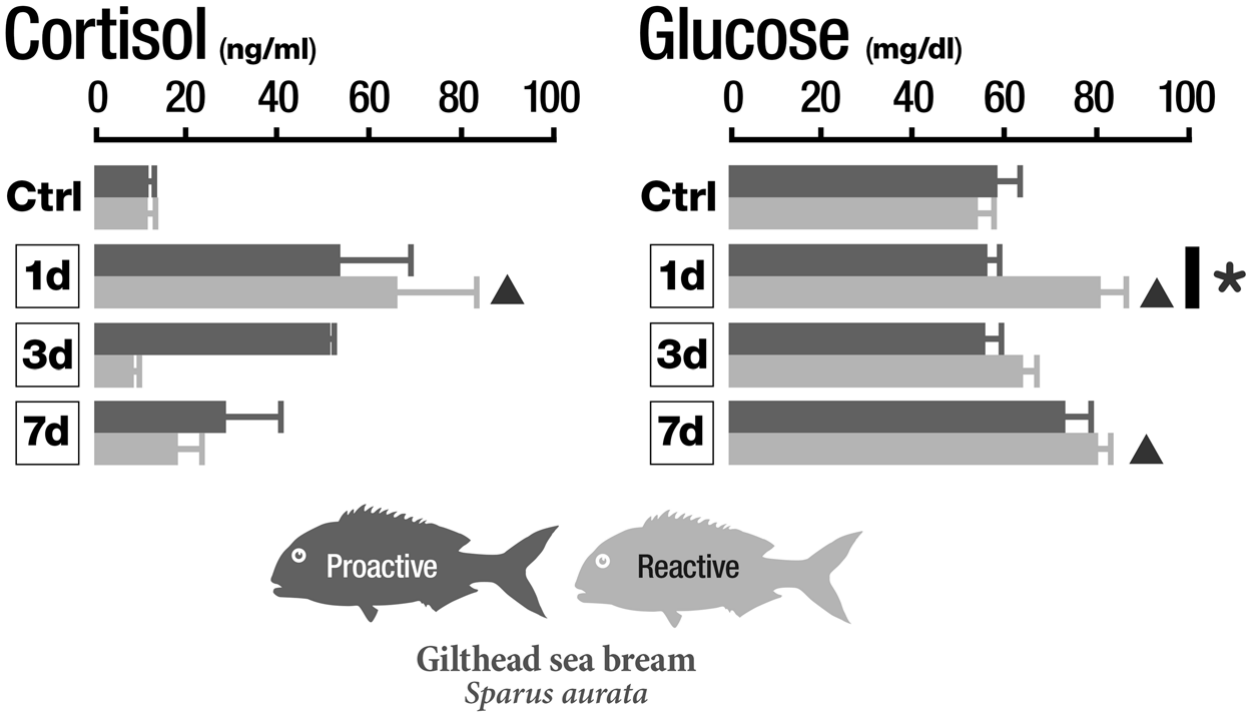
Plasma cortisol and glucose dynamics of proactive and reactive fish exposed to *Vibrio anguillarum* bacterin at 1, 3 and 7 days post-vaccination. *indicates significant differences between proactive and reactive fish (p < 0.05). ▴ indicates significant differences between reactive and control fish (p < 0.05). Data are presented as mean ± SD.

Due to the scarcity of sample amount associated to the small size of the fish and the fact that the endocrine stress response was assessed at short and medium time, but not immediate, catecholamines were not analyzed. One day after bath vaccination, significantly higher plasma cortisol levels were observed in the reactive group of fish, when compared with its respective control group (Fig. 4). For all other groups and conditions plasma cortisol was at control levels. Glucose levels in plasma were unaltered for all the conditions in proactive fish, but follow the same pattern as plasma cortisol response in reactive fish, i.e., a significant increase for day 1 post-vaccination (dpv) both when compared to the control group. A significant difference in glucose levels was observed between proactive and reactive groups at 1 dpv. Moreover, plasma glucose levels were significantly increased in the reactive group at 7 dpv, when compared with its respective control (Fig. 4).

## Discussion

The infectiveness of *Vibrio anguillarum* varies with the serotype and the characteristics of the host. In fish, once attached to mucosal external surfaces, septicemic infection progresses aggressively and can be lethal at 5 days post-attachment^20^, rendering the affected fish more vulnerable to infection by other opportunistic pathogens. The serotypes used in our study (O1, O2α and O2β) yield the highest pathogenicity^19^, and thus are an adequate insult to test the responsiveness of gills and liver to *Vibrio* bacterin. Vaccination in fish acts both as a preventive set of measures to elude seasonal or occasional pathogenic outbreaks and as a mean to guarantee sustainable and bio-secure aquaculture practices. However, pathogen-specific tailored vaccines can protect against disease but they may not prevent the spreading of the pathogen, which depends strongly on the characteristics of the host infection phenotype^21^: the outcome of a particular disease combines the whole set of the host’s physiological responses against the peculiarities of the pathogen’s infectiveness and spreading dynamics. Therefore, the inter-individual behavioural differences, and the overall performance of the individuals in a population, emerge as a proxy for underlying polarized neuro-immune-endocrine responses to stressors^22,23^. Selection for high or low immune responders to common pathogens may influence the likelihood of host-pathogen outcome. In the present study, proactive and reactive gilthead sea bream responded to *V. anguillarum* bacterin vaccination following two distinct phenotypes: high immune responders, and low immune/high metabolic responders, respectively.

In fish, gills and liver can be considered two different models in terms of immune response dynamics. Gills are a multifunctional organ, burdened with respiratory, osmoregulatory and defensive responses that harbours populations of infiltrated leukocytes inside a mucous matrix. These labelled gill-associated lymphoid tissues (GIALTs) construct an effective short-term immune response that acts locally but may expand systemically if not resolved in the first hours/days postinfection^24^. The liver is also a multifunctional organ, involved in metabolism regulation, xenobiotic clearance and medium-to-long term responsiveness to immune activation. In this sense, we analysed both organs in *S. aurata* as probes to evaluate the local and systemic medium-term effects (7 days) to bath vaccination with *V. anguillarum* bacterin. The gills appeared to be the most affected organ in terms of immune-related transcript expression. Proactive but not reactive fish showed a strong proinflammatory reaction headed by high (up to 10-fold) levels of *il1β* gene transcripts that remained elevated (up to 4-fold) 7 days post-vaccination (dpv). Another proinflammatory marker, *tnfα*, also showed an enhanced transcript expression (up to 2-fold at 7 dpv). The transcript *sod* presented an increased expression (up to 3-fold at 7 dpv) only in proactive fish throughout the experimental period. Both *il1β* and *tnfα* cytokine transcripts have been described as *bona fide* clues to inflammatory onset in response to pathogen infection and environmental stressors in behavioural-selected fish^25^. Superoxide dismutase’s, catalases and glutathione peroxidase’s quench reactive oxygen species (ROS) released by phagocytes to kill pathogens (the so-called respiratory or oxidative burst) during inflammatory reactions. The enhanced expression of *sod* transcripts may indicate, together with the minor differences observed in *gpx1*, *cat* and TOS, an increase of gill metabolism driven by the unfolding immune responses and also a regulatory response to the secretion of ROS such as superoxide anion and hydrogen peroxide by phagocytes^26^. This suggests that the long-lasting inflammatory process described for bath vaccinated sea bream are characterized by the recruitment of cellular populations of innate (early) immune defence (neutrophils and macrophages) in gill tissue.

Maintaining a sustained inflammatory response is energetically expensive, and can be considered a stressful situation by itself^27^. The systemic effects of such metabolic burden did not affect the expression of proinflammatory cytokine transcripts, nor biochemical and metabolic endpoints in the liver of proactive or reactive *S. aurata*. However, reactive but not proactive fish increased up to 2-fold the expression of hepatic antioxidant-related transcripts (*gpx1* and *cat*) and, notably, *ghr1* in a time-dependent fashion, with values peaking 7 dpv. The time-dependent enhancement of anti-oxidative transcripts and the peak of ALT values for reactive fish at 7 dpv suggest that reactive gilthead sea bream endured a low level but persistent systemic activation of immune components one week post-vaccination with *V. anguillarum* bacterin. *S. aurata* possess two copies of growth hormone (GH) receptors, being the *ghr1* transcript the most actively transcribed in the liver^28^. Cortisol, the main mediator of stress responses in fish, has been said to upregulate hepatic *ghr1* in gilthead sea bream, suggesting a role on fish growth^29^, which correlates with the observed elevation of plasma cortisol values in reactive *S. aurata* at 1 dpv.

Cortisol has been shown to suppress immune activation in stressed fish^27^, which may help to partly explain the lack of transcript expression of proinflammatory cytokine transcripts in reactive *S. aurata* as well as the sustained highs levels of cortisol in proactive fish compared to reactive sea bream in the first stages post-vaccination. As discussed above, *Vibrio* vaccination induces the expression of local proinflammatory responses in gills that seem to be regulated by increased plasma cortisol secretion, thus temporarily transforming an otherwise “proactive reaction” (i.e. low basal cortisol levels and low cortisol reactivity in stressful situations) into a reactive one during the initial stages (1 to 3 dpv) of vaccine exposition. This should be taken into account when designing a programmed vaccination before seasonal *Vibrio anguillarum* outbreaks.

In fish, the GH/insulin-like growth factor (IGF)-system is considered a stimulator of immune responses^30^, and a decline in the expression of pituitary GH transcripts has been described during vibriosis in *Sparus sarba*^31^, implying a reduction of growth in infected fish. However, the role of the GH/IGF system in fish remains controversial, and the administration of exogenous GH may enhance the cellular immune responses or have no effect in fish challenged with virulent pathogens^32–34^. Taken together, our results suggest that the liver of vaccinated reactive gilthead sea bream suffers from low-level immune activation and a metabolic over compensatory response.

Recently, we have demonstrated a strong species-specific proinflammatory reaction to bath vaccination with *Vibrio* bacterins in *S. aurata* mucosal immune tissues^35^ that matches the described here in the gills of proactive gilthead sea bream. In this light, a highly responsive *il1β*-driven inflammatory response seems to define the behavioural infection phenotype of gilthead sea bream exposed to waterborne *Vibrio anguillarum*. The proneness of proactive sea bream to elicit local pro-inflammatory responses in branchial tissues at the initial stages of vaccination may help to reduce the impact of vibriosis among the population, both in natural and artificial environments. However, the onset of inflammatory responses may also affect the physiological and metabolic trade-offs that sustain immunity activation, impairing the overall response to infection. The persistence, for extended periods of time, of *Vibrio* in flesh and water flows of aquaculture systems^36^ could also be partially avoided if an effective and strong defensive reaction starts in the peripheral mucosal immune tissues of farmed fish. Conversely, the low-immune/high-metabolic responder phenotype of reactive sea bream underpin a behavioural style of coping with virulent or vaccine attenuated diseases that may modulate the pacing of the infection, growth rates, competitive food intake and reproductive fitness. To date no studies have addressed the prevalence of a particular infection phenotype in natural environments in fish, but selecting for coping styles in controlled and farmed systems may help to define the effect of individuality/personality in the homeostatic regulation, the energetic and metabolic responses to the allostatic load and the covariation of behavioural traits with immune individual profiles.

## Methods

### Fish husbandry

*S. aurata* fry (N = 192) with an average weight of 6.49 ± 1.81 g (mean ± SD) were obtained from the fish farm Bersolaz-Culmarex (Puerto de Sagunto, Spain). Upon arrival to the fish facility AQUAB (Universitat Autònoma de Barcelona, Spain) fish were kept in a 1000 L tank (recirculating flow-through system) and acclimated for one month prior to the beginning of the coping styles screening tests. Fish were held in saltwater (34.4 ± 1.6), under controlled temperature of 21.9 ± 2.2 °C, in a 14 h light: 10 h dark photoperiod. These conditions were kept throughout the experiments. Oxygen was monitored daily. Ammonia, nitrites, nitrates and pH were monitored once a week. Fish were fed *ad libitum* every day at the same hour with a commercial diet, up to 24 h before the beginning of the vaccination assay and 24 h before each sampling moment. The experiment complied with the Guiding Principles for Biomedical Research Involving Animals (EU2010/63), the guidelines of the Spanish laws (law 32/2007 and RD 53/2013), and authorized by the Ethical Committee of the Universitat Autònoma de Barcelona (Spain) for the use of laboratory animals.

### Behavioural screening

Twelve hours before the beginning of the behavioural tests, groups of 12 fish were transferred from the housing tank to the experimental tanks in the behaviour room (23 L, 30 cm long x 28 cm wide × 28 cm deep). The experimental tanks were covered with white paper in order to reduce stress. Three individual coping style screening tests were performed (Fig. 1): (1) Net restraining test was used to discriminate between behavioural phenotypes and consisted in holding each fish suspended on a net (16 cm long × 12 cm wide) out of water for one min. The following behaviours were observed and registered for each individual: *latency to escape* (time in seconds for each fish to attempt scape, attempted escape defined as raise of the fish’s body from the net), *number of attempted escapes* and *time* (seconds) *between the first and last attempted escapes*. Criteria used to distinguish between behavioural phenotypes are shown in Fig. 1. (2) Open field test: Test tank was a 33 L (48 cm long × 24 cm wide × 29 cm deep) glass tank. Three sides of the tank were fully covered with white paper, in order to decrease stress and isolate from outer surroundings, leaving the fourth side for observation. A refuge area was habilitated in one of the tank’s ends, covering one third of the tank’s length and one third of its height with a black PVC division/separator. The remaining open space inside the tank was considered as the new environment. On the observer’s side, a horizontal line dividing the tank in two identical size halves (top and bottom) was painted. Fish were individually transferred to the refuge area in the tank and left there for 10 mins, in order to mitigate handling stress. After these 10 mins, the PVC division was carefully removed, starting a 10 min observation period. The following behaviours were observed and registered for each individual: *latency* (seconds) *to reach the upper half of the tank* (entrance into the area was considered when the animal’s cephalic region entered the tank’s upper half), *time* (seconds) *spent in the upper half of the tank* and *freezing time* (seconds). Freezing was defined as complete absence of movement for one second or more, not considering gills and eyes. *Ventilation rate*. Ventilation was calculated by counting the amount of seconds each fish needed to complete a total of 20 consecutive opercular or buccal movements. This was visually estimated as the fish were transferred to the refuge in the open field test. Ventilation rate was determined during 3 consecutive minutes and the average values were used in data analysis. (3) Novel object test: Test was initiated immediately after open field test, using the same tank. Three sides of the tank were covered with white paper, in order to decrease stress and isolate from outer surroundings, leaving the fourth side for observation. A multiple coloured Lego^®^ column (blue, green, red, yellow) (8.4 cm long, 3 cm wide, 22.6 cm deep) was used as novel object, and placed on one of the tank ends. Two vertical lines, at a distance of 10 and 5 cm from the novel object each, were painted on the remaining uncovered tank wall. Observation began immediately after the novel object was placed inside the tank, and lasted 10 mins. The following behaviours were observed and recorded: *latency* (seconds) *to enter the 10* *cm area, latency* (seconds) *to enter the 5* *cm area* (fish were considered to have entered an area once the animal’s cephalic region entered the said area) and *freezing time* (defined as complete absence of movement for one second or more, not considering gills and eyes).

### Fish vaccination

Gilthead sea bream were vaccinated with ICTHIOVAC® VR (Hipra, Spain), an inactivated vaccine against Vibriosis containing the formalin-killed *Vibrio anguillarum* serotypes O1, O2α and O2β. Proactive and reactive fish were vaccinated by bath immersion (diluted 1:10) during 1 min according to manufacturer’s instructions. A sham vaccinated group served as control group (time 0 h). After each procedure, fish were immediately placed on separated 300 L tanks. Eight experimental groups (n = 10 each group) were evaluated: 4 groups for reactive fish, and 4 groups for proactive fish. Fish were sampled 0, 3 and 7 days post-vaccination. Animals were anesthetized with tricaine methanesulfate, MS222 (1 g/L), the caudal fin was cut and blood was immediately withdrawn with heparinized capillary tubes. Blood was kept on ice, and posteriorly centrifuged at 2,500 rpm for 10 min for plasma isolation. Liver and gills were sampled, flash frozen in liquid nitrogen and kept at −80 °C until analysis.

### Transcriptional analysis

Total RNA was extracted from liver and gills using TRI Reagent^®^ and following manufacturer’s recommendations. RNA quantification was done using a NanoDrop Spectrophotometer (Thermo Fisher Scientific, USA) and RNA quality checked with Experion, using the Experion Standard Sens RNA chip (Bio-Rad Laboratories, USA). Reverse transcription was performed using 1 μg of RNA as a template with iScript™ cDNA synthesis kit (Bio-Rad, USA) according to manufacturer’s instructions. Efficiency of amplification was determined for each primer pair using serial 5-fold dilutions of pooled cDNA and calculated as E = 10(−1/s), where s is the slope generated from the serial dilutions. RT-qPCR was run in a Bio-Rad CFX384 Real-Time PCR Detection System (Bio-Rad, USA). Reactions were done using iTaqTM Universal SYBR® Green Supermix (Bio-Rad, USA) according to manufacturer’s instructions. Briefly, 1 cycle at 95 °C for 5 min, 40 cycles at 95 °C for 30 s, 60 °C for 30 s, and 72 °C for 30 s were run; samples were performed in triplicates. Expression data, obtained from three independent biological replicates, was used to calculate the threshold cycle (Ct) value. After checking primers’ efficiency, RT-qPCR analysis (Table 1) of all the individual samples was determined following the same protocol described above. *NormFinder* application was used to evaluate the most appropriate housekeeping gene among three: elongation factor-1α (*ef1α*); 18 s ribosomal RNA gene (18 *s*) and tubulin (*tub*). In liver, stability values of the candidate housekeeping genes were 0.114 for *ef1α*, 0.136 for *tubulin*, and 0.269 for 18 *s*. In gills, stability values of the candidate housekeeping genes were 0.091 for *ef1α*, 0.131 for *tubulin* and 0.234 for 18 *s*. Accordingly, the expression of the target genes was normalized using the best housekeeping gene *ef1α*, and gene expression calculated with the ΔΔCt method^37^. Relative normalised gene expression data is represented in the Fig. 2 and 3.

**Table 1:** Primers used for gene expression analysis in *S. aurata*.

Gene name	Acronym	Accession no.	Forward	Reverse
Elongation factor-1α	*ef1α*	AF184170	CCCGCCTCTGTTGCCTTCG	CAGCAGTGTGGTTCCGTTAGC
18S ribosomal RNA gene	18*s*	AY993930	GCATTTATCAGACCCAAAACC	AGTTGATAGGGCAGACATTCG
β-Actin	*β-actin*	X89920	TCCTGCGGAATCCATGAGA	GACGTCGCACTTCATGATGCT
Glyceraldehyde 3-phosphate dehydrogenase	*gapdh*	DQ641630	TGCCCAGTACGTTGTTGAGTCCAC	CAGACCCTCAATGATGCCGAAGTT
Interleukin 1β	*il1β*	AJ277166.2	TCAGCACCGCAGAAGAAAAC	TAACACTCTCCACCCTCCAC
Tumour necrosis factor- α	*tnfα*	AJ413189.2	TCGTTCAGAGTCTCCTGCAG	AAGAATTCTTAAAGTGCAAACACACCAAA
Immunoglobulin M	*igm*	JQ811851.1	GATCGTGACATCGTCTGAGG	TGTTGGGTTGTGGTTGTAGG
Glutathione peroxidase 1	*gpx1*	DQ524992	GAAGGTGGATGTGAATGGAAAAGATG	CTGACGGGACTCCAAATGATGG
Catalase	*cat*	JQ308823	TGGTCGAGAACTTGAAGGCTGTC	AGGACGCAGAAATGGCAGAGG
Superoxide dismutase	*sod2*	JQ308833	CCTGACCTGACCTACGACTATGG	AGTGCCTCCTGATAT TTCTCCTCTG
Lipoprotein lipase	*lpl*	AY495672	CGTTGCCAAGTTTGTGACCTG	AGGGTGTTCTGGTTGTCTGC
Growth hormone receptor type I	*ghr1*	AH014067.4	CACGTACTGGCTCCGTCTC	GCCGCTTTCCTGTTGTCAAG
Growth hormone receptor type II	*ghr1*	AY573601.2	GACCCCGAACTGCTCAAGAA	TTGTCGCTTTGCTCCTCGAT

### Biochemical analysis in liver and gills

Liver and gills samples were processed as previously described^38^. Glucose, cholesterol, triglycerides, ALT, ALP and AST were determined using commercial reagents (Beckman Coulter, Beckman Coulter Irland Inc. Ireland; Olympus Systems Reagents, Hamburg, Germany), following manufacturers indications. EA was analysed by measuring the hydrolysis of p-nitrophenyl acetate to p-nitrophenol as described elsewhere^39^, with some modifications^38^. TOS was measured based on the reaction that ferric ion makes a coloured complex with xylenol orange in an acidic medium^40^, with some modifications^38^. TAC was determined based on 2,20-azinobis-(3-ethylbenzothiazoline-6-sulfonate) decolourization by antioxidants according to their concentrations and antioxidant capacities as described previously^40^, with some modifications^38^. All the methods were performed with an automatic analyser (AU 600 automated biochemical analyser, Olympus, Minneapolis, USA) and all methods were previously validated for fish samples. All biochemical results are expressed per mg of protein.

### Cortisol and glucose analysis in plasma

Plasma cortisol levels were measured by radioimmunoassay and radioactivity was quantified using a liquid scintillation counter (Scintillation Counter Wallac 1409, PerkinElmer). The anti-cortisol antibody (ref. 07-121016, MP Biomedicals, Solon, OH, USA) was used for the assay at a final dilution of 1:4500. The lower detection limit of the assay was 0.16 ng/mL. Plasma glucose was determined by enzymatic colorimetric analysis in Enzyme-Linked ImmunoSorbent Assay (ELISA) plates using commercial kits (Biomérieux, France).

### Statistical analysis

Behavioural tests results were checked for normality and homogeneity of variance applying Shapiro-Wilk’s and Levene’s tests, respectively. Behavioural variables showed a not normal distribution. Both consistency through contexts (correlation between/among behaviour tests) and consistency between ventilatory rate and behavioural tests were assessed using Spearman’s correlation coefficient. Two-way ANOVA tests were used to determine differences between personality groups and vaccine treatments in plasma and biochemical parameters as well as in gene expression; results are expressed as average ± SD (standard deviation). These analyses were followed by post-hoc Bonferroni test, in order to identify possible differences between groups. Said differences were established as α < 0.05. All statistical analyses were performed using STATISTICA 7 (StatSoftV7**®**) and Graph Pad Prism V.6.1. Software.
